# Identification of Conditions for Successful Aphid Control by Ladybirds in Greenhouses

**DOI:** 10.3390/insects8020038

**Published:** 2017-03-28

**Authors:** Eric W. Riddick

**Affiliations:** National Biological Control Laboratory, Jamie Whitten Delta States Research Center, Agricultural Research Service, USDA, Stoneville, MS 38776, USA; eric.riddick@ars.usda.gov; Tel.: +1-662-686-3646; Fax: +1-662-686-5281

**Keywords:** Aphididae, biological control, Coccinellidae, organic agriculture, pest management, predation

## Abstract

As part of my research on the mass production and augmentative release of ladybirds, I reviewed the primary research literature to test the prediction that ladybirds are effective aphid predators in greenhouses. Aphid population reduction exceeded 50% in most studies and ladybird release rates usually did not correlate with aphid reduction. The ratio of aphid reduction/release rate was slightly less for larvae than adults in some studies, suggesting that larvae were less effective (than adults) in suppressing aphids. Some adult releases were inside cages, thereby limiting adult dispersion from plants. Overall, the ratio of aphid reduction/release rate was greatest for ladybird adults of the normal strain (several species combined), but least for adults of a flightless *Harmonia axyridis* strain. The combined action of ladybirds and hymenopteran parasitoids could have a net positive effect on aphid population suppression and, consequently, on host (crop) plants. However, ladybird encounters with aphid-tending or foraging ants must be reduced. Deploying ladybirds to help manage aphids in greenhouses and similar protective structures is encouraged.

## 1. Introduction

Culturing plants in greenhouses, glasshouses, or hothouses has existed in Europe and Asia since the early 19th century, with expansion into North America and other regions of the world in recent years [1,2,3]. Despite the protective, semi-enclosed environment in greenhouses, a number of herbivorous pests routinely invade them and infest crop and non-crop plants. Some of the traditional pests of importance in greenhouses include spider mites, whiteflies, thrips, and aphids, all of which have great potential to reach outbreak densities and result in production losses, if not controlled [4].

Of the approximately 4700 species in the Family Aphididae, nearly 100 are significant agricultural pests [5]. In greenhouses, the most important species attacking vegetable crops are *Aphis gossypii* Glover, *Macrosiphum euphorbiae* (Thomas), *Aulacorthum solani* (Kaltenbach), and *Myzus persicae* Sulzer [6]; those attacking ornamentals are *A. gossypii*, *M. euphorbiae*, *A. solani*, *M. persicae*, *Brachycaudus helichrysi* (Kaltenbach), *Macrosiphoniella sanborni* (Gillette), and *Macrosiphum rosae* (L.) [7]. Important species attacking small fruits (e.g., strawberries) in greenhouses are *Chaetosiphon fragaefolii* (Cockerell), *A. gossypii*, *M. euphorbiae*, *M. persicae*, and *Myzus ascalonicus* Doncaster [8]. These species can have a dramatic impact on crop production via direct feeding injury to crop plants, the transmission of plant viruses between individual plants, and the rapid development of resistance to insecticides [9,10,11]. Honeydew, excreted by aphids, adheres to plant foliage as well as some fruits (e.g., strawberries), and promulgates the growth of sooty mold. Sooty mold is unsightly and renders some fruit unsuitable for sale in traditional markets.

Due to their potential to rapidly develop resistance to insecticides, there is growing interest in using non-insecticidal control methods, such as biological control, to suppress aphids in greenhouses [12,13] and similar structures. Aphid parasitoids are sometimes used for this purpose [14]; in recent years, mass-reared hymenopteran parasitoids (Family Aphidiidae, and Family Aphelinidae) have become the option of choice, rather than predators [15,16]. However, there are several drawbacks to using aphid parasitoids. The cost of rearing aphid parasitoids for augmentative release in greenhouses can be high, because host plants and live hosts (aphids) must be supplied to maintain colonies in commercial mass production. There are only a few suitable factitious hosts or artificial media for aphids or developing parasitoids. Another problem is related to host plant defenses against aphid herbivory; glandular trichomes can greatly reduce parasitism rates and even increase parasitoid mortality rates [17]. Aphid parasitoids are commonly attacked by hyperparasitoids in open field and greenhouse settings [14]. Additionally, immature parasitoids often succumb to intraguild predation from generalist predators, such as ladybird beetles, i.e., lady beetles (Family Coccinellidae), but net consequences of intraguild predation could be positive or negative to aphid suppression in open field situations [16,18].

Ladybird beetles are common biological control agents of aphids in natural field settings [19,20,21,22,23,24,25,26,27,28]. Many researchers claim that ladybirds are incapable of regulating aphid populations under natural field conditions for reasons relating to their voracity, search efficiency, predation capacity, and reproductive rate [29,30,31,32,33], but there are a few reported examples of apparent regulation [34,35]. Nevertheless, the ability to regulate aphid populations is not essential if repeated (inundative) releases of ladybirds into a greenhouse delays or prevents aphid outbreaks. Historical evidence suggests that several ladybird beetle species have the potential to reduce aphid populations in greenhouses or glasshouses [36,37]. We consider the ladybird beetle’s ability to cause rapid declines in aphid population density (and thereby diverting aphid outbreaks), via repeated ladybird releases or increasing the ladybird release rate, to be an alternative gauge of effectiveness [38,39] in greenhouses and glasshouses.

The efficacy of ladybirds as aphid predators in greenhouses and glasshouses has been reviewed previously [40], but this current study represents a more comprehensive review of the published literature using the United States Department of Agriculture (USDA), National Agricultural Library, Digitop Literature Database (Navigator platform) to assess the abstracts that include the search terms “Coccinellidae and greenhouse” or “Coccinellidae and glasshouse”. The Navigator platform is associated with the following literature databases: AGRICOLA, AGRIS, BIOSIS Previews, CAB Abstracts, Fish & Fisheries, GEOBASE, EBSCO Environment, MEDLINE, Scopus, Web of Science, Wildlife and Ecology, FSTA, Treesearch, and Zoological Record.

**Figure 1:**
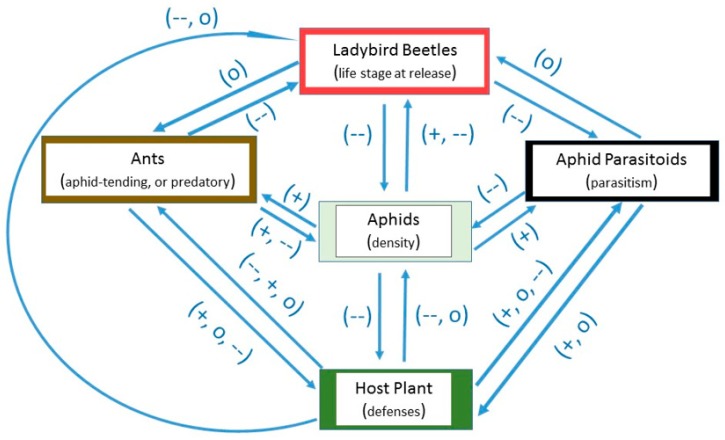
Generalized schematic of the positive (+), neutral (o), or negative (--) interactions that could occur between host plants, aphids, ladybirds, aphid parasitoids, and ants in greenhouses.

In this review, the prediction that ladybird beetles can be effective predators of aphids in greenhouses and glasshouses was tested. Biotic factors including host plant defenses, ladybird life stage at release, interactions with other predators, aphid parasitoids, and ants could limit ladybird effectiveness. A generalized schematic of the positive (+), neutral (o), or negative (--) interactions that could arise between these factors is presented in Figure 1. Prior research conducted primarily in the laboratory and in open field settings suggests that host plant defenses could have negative or neutral effects on aphids as well as ladybirds [41]. Also, aphid density has a positive effect on ladybirds [21,22], but the effect can become negative if prey becomes scarce, resulting in starvation or cannibalism amongst immature ladybirds. Aphid parasitoids have negative effects on their aphid hosts [14,16]. Ladybirds can have negative effects on aphid parasitoids developing inside aphid hosts, through aphid predation [18]. Aphid-tending ants could have positive effects on aphids which they tend [42], but negative effects on most species of aphidophagous ladybirds [43]. Foraging predatory ants, which kill rather than tend aphids, could have negative effects on aphid density and on the predation potential of ladybirds [44].

Relevant studies providing data on ladybird releases were tabulated for an assessment of the capacity of ladybirds to reduce aphid populations. Percent aphid reduction was calculated using one of two methods: (1) When control cages or control greenhouses were used, and aphid density was equilibrated at the onset of the experiment, percent aphid reduction was calculated from aphid density on control and test plants [(control − test)/control (×100)] at post-release evaluation; (2) In the absence of controls, or if aphid density was variable at the onset of the experiment, percent aphid reduction was calculated from aphid density on plants before releasing ladybirds (pre-release) and after releasing them (post-release) [(pre-release − post-release)/pre-release (×100)].

To compare ladybird efficacy across studies listed in Table 1, I used the Pearson Product Moment Correlation to determine if release rate and aphid population reduction were correlated. A Student’s *t*-test compared the ratio of aphid population reduction/release rate between larvae and adults. Correlations and mean values were significantly different when *p* < 0.05. SigmaStat 3.0.1 (interfaced through Sigma Plot 12, SAS Institute Inc., Cary, NC, USA) software assisted with data analysis.

## 2. Factors Potentially Affecting Ladybird Success in Greenhouses

### 2.1. Host Plant Defenses

Defenses that plants deploy to reduce herbivory can alter the effectiveness of ladybird beetles [41]. Foliage of faba bean *Vicia faba* L. 79S4 cultivar (partially resistant) reduced the reproductive rate of the black bean aphid *Aphis fabae* Scopoli in pots enclosed in cages [45]. Although the mechanism of resistance was not indicated, perhaps allelochemicals in cultivar 79S4 foliage reduced *A. fabae* herbivory. Allelochemicals are secondary plant compounds that are sometimes toxic to aphids, and thereby reduce herbivory. These compounds may or may not affect predation capacity of ladybirds which consume aphids on defended plants. A release ratio of 1:1 (1 *Coccinella septempunctata* L. neonate larva: 1, two-day old *A. fabae* adult) per plant, reduced aphid density by 57.1% on the partially resistant cultivar, but just 33% on the susceptible cultivar (*V. faba*, cv. major) in nine days, when compared against control plants, caged plants without ladybird beetles (Table 1). This observation suggests that the combination of partial *V. faba* resistance and *C. septempunctata* predation was more effective in reducing *A. fabae* density than either method alone [45]. Thus, host plant defense did not reduce predation capacity of *C. septempunctata* in this study.

Morphological plant defenses, such as the degree of “waxiness” on the leaf surface, can affect predation potential of ladybirds foraging on plants. For example, adults of the convergent lady beetle *Hippodamia convergens* Guérin-Ménéville foraged more effectively for pea aphids *Acyrthosiphon pisum* (Harris) on caged pea *Pisum sativum* L. plants with a reduced leaf wax phenotype than on those with a normal leaf wax phenotype [46]. Waxy leaves may impede the movement of *H. convergens* on plants in cages, thereby reducing its effectiveness as an aphid predator.

Plant epidermal hairs (i.e., plant trichomes) can alter the foraging behavior of coccinellids on plants and are known to impale or trap some aphid species [41]. Trichomes may or may not affect predation capacity of ladybirds which forage for aphids on defended plants. Coccinellid adults (e.g., *H. convergens*) spent less time foraging on potted wild potato *Solanum berthaultii* Hawkes containing a high density of glandular trichomes on its leaves and stems than on an interspecific hybrid of cultivated potato *Solanum tuberosum* L. x wild potato *S. berthaultii* F_3_, containing a moderate density of glandular trichomes, or on *S. tuberosum*, containing no glandular trichomes [47]. Thus, *H. convergens* predation capacity was presumably reduced on potato foliage containing high trichome density.

A reduction in foraging time on plants containing trichomes can result in a reduction of aphid control, as shown for 2nd instar larvae. Note that 2nd instar larvae of the ladybird *Coleomegilla maculata* DeGeer did not remain on potted cucumber *Cucumis sativus* L. plants long enough to suppress densities of the melon/cotton aphid *A. gossypii* [48]. Glandular trichomes on *C. sativus* leaves were perceived as being “irritating” to *C. maculata* larvae. The effect of trichomes on ladybird larvae is species specific. In the same study, *C. sativus* glandular trichomes did not negatively affect predation capacity of 2nd instar larvae of other ladybirds, such as *Cycloneda sanguinea* (L.) and *Adalia bipunctata* (L.). Aphid populations were reduced by 50.4% and 36.0% in 11 days by *C. sanguinea* and *A. bipunctata*, respectively. Thus, cucumber trichomes have differential effects on ladybird larvae; *C. maculata* were affected negatively, but *C. sanguinea* and *A. bipunctata* were not.

The Antares, CNPA 7H, or DeltaOpal cultivars of cotton *Gossypium hirsutum* L., representing plants with glabrous (low), hirsute (moderate), and pilose (high) trichome density, did not reduce the predation capacity of *C. sanguinea* and *H. convergens* [49]. Young plants (approximately 30 days old) were infested with 100 aphids (*A. gossypii*) and two days later, a single *H. convergens* or *C. sanguinea* adult female was released onto infested plants. *H. convergens* and *C. sanguinea* females reduced the aphid population on plants by an average of 87% and 93.5%, respectively, regardless of trichome density and within two days of release, in comparison to aphid density on plants not inoculated with a ladybird.

**Figure 2:**
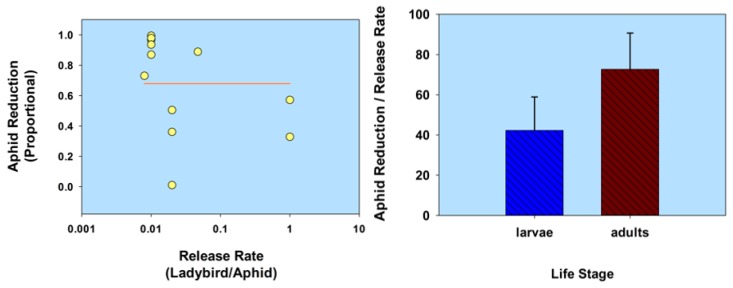
Scatterplot of release rate vs. aphid population reduction and bar graph of ladybird life stage vs. the ratio of aphid reduction/release rate, in relation to host plant defenses. The release rate is plotted on a common logarithmic scale. Data based on five studies and 12 observations (see Table 1).

To summarize this section, host plant defenses can affect the ability of ladybirds to reduce aphid population density in greenhouses. However, the negative reports are occasional as mentioned herein. Based upon a comparative analysis of the data listed in Table 1 for this section (*Host plant defenses*), aphid population reduction did not correlate with release rate (*r* = −0.34; *p* = 0.28; *n* =12); and ladybird larvae were not significantly less effective than adults, based on a ratio of aphid reduction/release rate (*t* = 1.13; df = 10; *p* = 0.29; Figure 2; Table 1.

### 2.2. Life Stage

#### 2.2.1. Normal Strain

Life stage and release rate could affect the ability of ladybirds to suppress aphid populations. In a manipulative experiment involving potted chrysanthemums (*Chrysanthemum indicum* L. cultivar BGA Tuneful), 2nd instar larvae of three species, *C. maculata*, *A. bipunctata*, and *C. sanguinea* reduced the density of *M. persicae* [48]. For example, at a release density of 10 larvae (2nd instars) per replicate pot, with approximately 1000 *M. persicae* per pot (four plant cuttings per pot), the density of aphids was reduced by 97%, 98%, and 99% in seven days, due to predation by *C. maculata*, *A. bipunctata*, and *C. sanguinea*, respectively (see Table 1). In the control pots (with approximately 1000 aphids per pot, without ladybird larvae), *M. persicae* density increased 2.1-fold in seven days. This study demonstrates the potential benefits of using ladybird larvae. Ladybird adults tended to readily depart from plants soon after being released. Therefore, predation potential of one species, *A. bipunctata*, was estimated in cages. When compared against control cages, *A. bipunctata* adults in test cages were highly effective, reducing *M. persicae* by 73.0% to 88.8% in two weeks. When mobility is restricted to cages, adult ladybirds are generally as effective as larvae in reducing *M. persicae* density.

In another study, 9.0 m^2^ plots were set up to test the efficacy of two ladybird beetle species, *C. septempunctata* and *A. bipunctata* [50]. A single release of 1st instar larvae of both species reduced aphid populations on crop plants. When aphid density was compared between control, commercial greenhouses (no predators released) and experimental greenhouses, both ladybird species curbed population growth of *M. persicae* on chrysanthemum (*Chrysanthemum morifolium* Ramat.) and sweet pepper (*Capsicum annuum* L.), respectively, in 8–10 days at several release rates. The release rate (number of ladybirds/ number of aphids) did not appear to have an effect on aphid reduction.

In a preliminary study, two ladybirds, *Lemnia biplagiata* (Swartz) and *Leis dimidiata* (F.), were tested for their capacity to reduce melon aphid (*A. gossypii*) numbers on cucumbers [51]. The authors stated that releases of 2nd instar larvae of *L. dimidiata* were more effective than adults, since adults tended to disperse from plants at low aphid density. The larvae continued to search foliage for aphids, even at low aphid density. For *L. dimidiata*, a release ratio of 1:10 or 1:20, ladybird: aphid was suitable to suppress *A. gossypii* populations by 85%–90%. Unfortunately, the authors didn’t provide information on *A. gossypii* density at release or post-release of *L. dimidiata*. Despite this, the authors recommend using both *L. dimidiata* and *L. biplagiata* to suppress aphid populations on cucumbers, peppers, and eggplants [51].

*Harmonia axyridis* (Pallas) larvae (274 3rd and 4th instars) and adults (726 overwintered adults) were released on strawberry (*Fragaria x ananassa* Duchesne) plants to reduce populations of two aphid species, *Aphis forbesi* Weed and *Chaetosiphon minor* (Forbes) [52]. In one week, aphid density (both species combined) decreased from nearly 1200 to 200 aphids per 20 plants. However, aphids began to rebound in a few weeks, because released larvae metamorphosed into pupae and then adults. Adults (both newly emerged and overwintered adults) then dispersed from the low-lying plants, leaving the greenhouse (since the greenhouse was open). Nevertheless, aphid densities decreased again as enough newly emerged adults remained in the greenhouse long enough to oviposit on plants; larvae hatching from these eggs helped reduce aphid densities. In another greenhouse at the same location and in the same season, *H. axyridis* larvae (750 3rd and 4th instars) and no adults were released onto strawberry plants. Aphid density decreased over 29 days from approximately 1300 to 200 aphids per 20 plants as a result of *H. axyridis* predation, suggesting that many of the larvae remained on the plants, fed on aphids, and pupated successfully; emerging adults contributed to aphid control as well. Note that the initial release rate was high (0.58 larvae/aphids), the time frame of the study was 29 days, and aphid reduction was 85%. Apparently, newly emerged *H. axyridis* adults were prevented from exiting the greenhouse.

In a study involving aphids on strawberry plants, in replicate (1 m^3^ nylon) cages, *C. maculata* 3rd instars showed promise in reducing the cotton aphid, *A. gossypii*, particularly at high population densities [53]. Two weeks after releasing *C. maculata*, *A. gossypii* densities were reduced by 87% and 96%, at a release rate of 0.20 and 0.33 ladybird/aphid density, respectively. At a lower release rate (0.07 ladybird/aphid), the *A. gossypii* population was reduced by 47%.

In another study, *H. axyridis* or *C. septempunctata* was released to manage primarily *A. gossypii* on strawberry plants [59]. The practice of removing “old” leaves from plants (i.e., cultural control) was useful in managing low density populations. At high densities, *H. axyridis* or *C. septempunctata* was released on leaves, flowers, and fruit [59]. Neither the life stage of the ladybird nor the quantity released was stated in this study. In a companion study, the researchers [60] released *C. septempunctata* (life stage not mentioned) to suppress *A. gossypii* and *Aphis craccivora* Koch on sweet pepper (*C. annuum*). Although the quantity or life stage of the ladybirds released was not specifically mentioned, the authors suggest that both ladybird species contributed to aphid population reduction in the test greenhouse in contrast to the control greenhouse (without ladybirds). Because of the lack of ladybird release rates, this study was not listed in Table 1.

The population density of *M. persicae* was reduced significantly by *H. axyridis* (presumably adults) on chile *Capsicum annum* L. plants held in screened cages [54]. In the experiment, plants were inoculated with *H. axyridis* and *M. persicae* at ladybird: aphid ratios ranging from 1:20 to 1:640 in replicated treatment cages (see Table 1). In comparison to control cages (lacking *H. axyridis*), aphid density was significantly reduced by at least 95% at all densities, except the 1:640 density (85.7%), over a 10-day sampling period. This study shows the potential of *H. axyridis* adults as a predator of *M. persicae* on chile plants. It is very unlikely that the same positive results would be achievable if chile plants were not enclosed in cages, because of the propensity of *H. axyridis* adults to fly away from plants, as documented in other studies.

Cucumber (*Cucumis sativus* L.) plants artificially infested with *A. gossypii* at a density of 50 aphids per plant were exposed to neonate larvae (emerging from egg clutches) of *H. axyridis* of a lab-cultured flightless strain, in replicate greenhouses, including a control greenhouse of identical dimensions (but no ladybird releases) [55]. Releases of 5 or 10 (rather than 1) egg clutches, containing an average of 20 eggs/clutch, per cucumber plant, proved effective, as 1st instar larvae hatched and consumed *A. gossypii* within five days post-release. By the eighth day, *A. gossypii* began to increase their densities again, suggesting that multiple releases of *H. axyridis* egg clutches and/or 1st instar larvae would be necessary to further reduce the aphid population.

#### 2.2.2. Flightless Strain

Both larvae and adults of a flightless strain of *H. axyridis* were tested as biological control agents against *M. persicae* and turnip aphid *Lipaphis erysimi* (Kaltenbach) on seedlings of non-heading *Brassica rapa* L. (2 cultivars) [56]. At an average initial density of 1.4–7.8 aphids per plant or 1.4 aphids per plant, release rates of 2 *H. axyridis* adults per m^2^ or 10 larvae per m^2^ area of plot (80 plants/plot) were sufficient to prevent significant increases in aphid densities over three weeks. In comparison to control plots (no releases), *M. persicae* density was 72%–98% lower and *L. erysimi* density was 81% lower in test plots with one release of *H. axyridis* adults. Flightless *H. axyridis* adults remained on experimental plants, in release plots, much longer than larvae [56].

In another study, 2nd instar larvae or adults of a flightless strain of *H. axyridis* were tested against *A. solani* on cultivated eggplants *Solanum melongena* (L.) [57]. A total of 600 larvae or 120 adults were released three times in the test plots, respectively; no predators were released in the control plots. This translated into 10 *H. axyridis* larvae per plant versus 2 adults per plant (60 plants per plot). Approximately 21 days after the third release of ladybird larvae and adults into separate test plots, 30 aphids per leaf (median value) were on plants in the control (no-release) plots, and 10 aphids per leaf (median value) were on plants in both test plots (larvae release plot, and adult release plot). Thus, *A. solani* population density was reduced by 66.7% in both test plots, when compared against the control plot (Table 1). Approximately 35 days after the third release of larvae and adults, *A. solani* density was reduced by 70% and 60% in the larval release and adult release plots, respectively, compared against the control plot [57]. Since the larval stage does not last more than 14 days at ambient conditions, much of the predation in the larval release plot beyond 21 days after the third release was certainly due to newly metamorphosed adults. The very slight decline in percent aphid reduction in the adult release plot, after 35 days post-release, was probably due to the decline in activity and death of some of these flightless adults.

**Figure 3:**
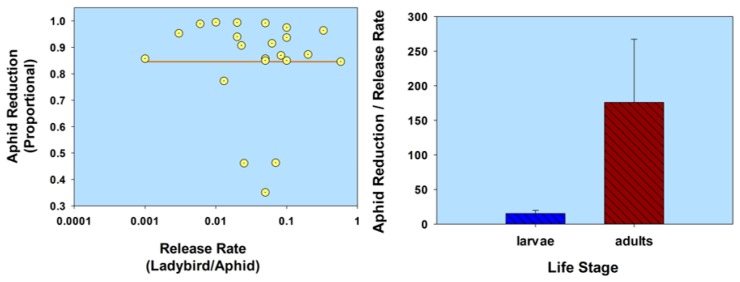
Scatterplot of release rate vs. aphid reduction and bar graph of ladybird life stage (normal strain) vs. the ratio of aphid reduction/release rate. The release rate is plotted on a common logarithmic scale. Data based on five studies and 22 observations (see Table 1).

**Figure 4:**
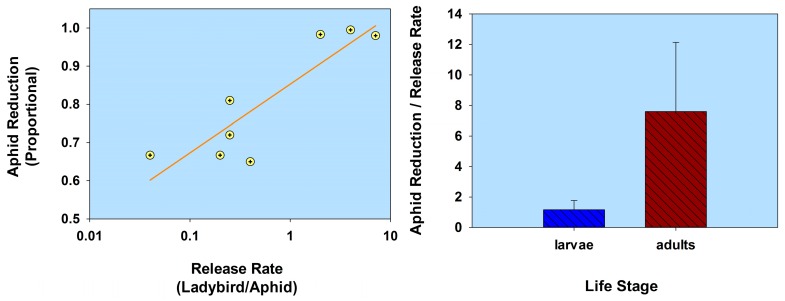
Scatterplot of release rate vs. aphid reduction and bar graph of ladybird life stage (flightless strain) vs. the ratio of aphid reduction/release rate. The release rate is plotted on a common logarithmic scale. Data based on three studies and 8 observations (see Table 1).

In summarizing this section (*Life stage*) using a comparative analysis of the results listed in Table 1, aphid population reduction didn’t correlate with ladybird (normal strain) release rate (*r* = 0.055; *p* = 0.81; *n* = 22); and ladybird larvae were less effective than adults, based on a ratio of aphid population reduction/release rate (*t* = 2.14; df = 20; *p* = 0.045; Figure 3; Table 1). Note that some of the adult releases were inside cages, thereby limiting adult dispersion from plants. In contrast, aphid population reduction was in fact correlated with the release of flightless *H. axyridis (r* = 0.79; *p* = 0.02; *n* = 8); and larvae were not significantly less effective than adults (*t* = 1.88; df = 6; *p* = 0.11; Figure 4; Table 1). None of the adults of the flightless strain were released into cages. Overall, the ratio of aphid reduction/release rate was greatest (exceeding a value of 150) for ladybird adults of several species of the normal strain, but least (less than a value of 8) for adults of a flightless *H. axyridis* strain. This suggests that adults of the normal strain could be more effective (than the flightless strain) in suppressing aphids. Further research is necessary to confirm these results, especially because the number of studies involving releases of the flightless strain are limited in this review.

### 2.3. Other Aphid Predators

The presence of other aphid predators on shared host plants could affect ladybird success, if ladybirds avoid these plants. If ladybirds forage on shared host plants, the interaction between predators could result in intraguild predation. Intraguild predation, in which one ladybird species attacks and kills another ladybird or non-ladybird predator, has been documented most often under laboratory conditions [61]. For example, *H. axyridis* larvae and adults are known to function as intraguild predators of other ladybirds and syrphid flies [61,62,63], although *H. axyridis* can occasionally serve as intraguild prey for syrphids [63]. Also, *H. axyridis* larvae are intraguild prey for lacewing *Chrysoperla carnea* (Stephens) larvae [64,65]. However, research suggests that intraguild predation usually does not affect the ability of predators to suppress pest densities in the field [66].

When intraguild predation is limited, or non-existent, ladybirds can reduce aphid densities in greenhouses in the presence of other aphid predators. The ability of *Coccinella transversoguttata* Faldermann adults to reduce *M. persicae* densities in the presence or absence of two hemipteran predators, *Geocoris bullatus* (Say) and *Nabis americoferus* (Carayon), was tested on caged sugarbeet, *Beta vulgaris vulgaris* L. [58]. *C. transversoguttata* adults reduced *M. persicae* populations when used alone or when combined with *G. bullatus, N. americoferus*, or *G. bullatus* adults. A release ratio of 4:114 (ladybird: aphid) resulted in a decline to less than 1 *M. persicae* per nine plants in six days. When 2 *C. transversoguttata* were combined with 4 *G. bullatus* or with 4 *G. bullatus* and 4 *N. americoferus*, it also resulted in a decline to less than 1 *M. persicae* in six days. Thus, aphid population reduction was 100% in all treatments (Table 1). In contrast, the control cages (without *C. transversoguttata* or any other predator species) contained an average of 416 *M. persicae* within the same time frame [58].

Undoubtedly, ladybirds and other aphid predators will occasionally come in contact with each other in greenhouses. Despite this fact, reliable quantitative data on the suppression of aphid populations under these conditions is scarce.

### 2.4. Aphid Parasitoids

The presence of aphid parasitoids on shared plants could affect the ability of ladybirds to reduce aphid densities. Aphid parasitoids and ladybirds typically interact when ladybirds come in contact with parasitized aphids. In the laboratory, ladybirds (*H. axyridis* and *C. septempunctata* larvae and adults) consumed un-parasitized aphids as readily as newly parasitized ones, but did not prefer consuming mummified aphids [67,68].

The frequency in which ladybirds prey on parasitized (and mummified) aphids in greenhouses is difficult to detect and quantify. The paucity of information on the net consequences of intraguild predation (of immature parasitoids) by ladybirds on aphid suppression in greenhouses signals a need for more research on this important topic. Nevertheless, the limited evidence suggests that the combined action of aphid parasitoids and ladybirds has a net positive effect on aphid population suppression (or limiting population growth) on plants. In a preliminary study, the convergent lady beetle *H. convergens* (10 adults/m^2^) and the parasitoid *Aphidius colemani* Viereck (adults at density of 1 adult/15 m^2^) were released in a glasshouse to suppress *A. gossypii* on strawberries [69]. Within one week, the combined effect of the two natural enemies controlled the aphid outbreak [69]. However, the authors did not provide the data on percent aphid reduction in this study.

The ladybird *H. axyridis* complemented an aphid parasitoid, *Aphelinus asychis* Walker, in suppressing *M. euphorbiae* on cut roses *Rosa hybrida* L. in a replicate cage and open-release (without cages) trial [70]. Larva and adult *H. axyridis* attacked *M. euphorbiae* and *A. asychis* mummies; *H. axyridis* larvae, not adults, showed a preference for killing and consuming aphids rather than mummies. In cages, *M. euphorbiae* peak densities were 75% lower when *H. axyridis* and mummies were present together, in comparison to cages with mummies alone. This cage study implies that *H. axyridis* complements the action of *A. asychis* despite the consumption of some of the parasitoid mummies. Note that *H. axyridis* adults escaped from the greenhouse after the first trial through a poorly fitting screen over the exhaust fan. After refitting the screen, a second release was more successful, with the establishment of adults which reproduced on plants. Soon after peak *H. axyridis* density was observed in the greenhouse, *M. euphorbiae* densities decreased more than 90% [70]. Perhaps the combination of ladybirds and parasitoids exerted an additive effect on aphid control. Note that the authors mentioned that caution must be used when interpreting the results of this study, as the greenhouse release was not replicated and it did not include a control greenhouse (where *H. axyridis* was not released).

Researchers released *C. septempunctata* larvae and adults to suppress primarily *A. gossypii* and *A. craccivora* on sweet pepper (*C. annuum*) [60]. In addition, they released the parasitoid *A. colemani* to suppress the aphid *M. persicae* in the same greenhouse. They found that *A. colemani* was capable of controlling low density populations of *M. persicae*. The authors did not indicate if *C. septempunctata* had a positive or negative effect on *A. colemani*. An ideal scenario would be the co-existence of ladybirds and aphid parasitoids with limited or no intraguild predation. The ability of aphid parasitoids to detect aphid aggregations in which a ladybird is foraging, or has been foraging, and to avoid those aggregations, has been revealed via laboratory and greenhouse cage experiments [71]. Perhaps adult aphid parasitoids are capable of detecting chemical signals left behind on foliage by ladybird beetles [72]. Females of three parasitoid species, *Aphidius eadyi* (Stary, Gonzalez, and Hall), *Aphidius ervi* (Haliday), and *Praon volucre* (Haliday), detected chemical trails on leaves visited by *C. septempunctata* and *A. bipunctata* [72].

In summarizing this section, ladybird predation of parasitized aphids (mummies) can occur on crop plants in greenhouses, but the net effect on aphid suppression is often positive or neutral, rather than negative. The actions of ladybirds and aphid parasitoids can increase the net negative effect on aphid population density. More research is necessary to convincingly demonstrate that the combination of ladybirds and parasitoids is more effective than either on their own for aphid suppression.

### 2.5. Foraging and Aphid-Tending Ants

The presence of ants on shared plants could affect the ability of ladybirds to reduce aphid densities. Antagonistic encounters between ants (Formicidae) and ladybirds have been reported most often on plants in open field conditions or in highly manipulated experiments in the laboratory. Foraging, predatory ants can negatively affect predation potential of ladybirds, when ants attack and kill larvae and deter adults from searching for aphids [73]. For example, workers of the red imported fire ant *Solenopsis invicta* (Buren) reduced the survival of ladybirds (*C. septempunctata* and *H. convergens* larvae) by 50% on cotton *G. hirsutum* plants in cages, in comparison to controls (cotton plants in cages without fire ants) [74].

The mutualism between some ant and aphid species has been recognized for decades. Ants tend aphids for their honeydew and aggressively ward-off ladybirds, foraging for aphids [42]. Thus, ants protect the aphids from ladybird predation [42,43,75,76]. The disruption of ladybird predation by aphid-tending ants [20,42] is a major concern. Published research to document the impact of aphid-tending ants on ladybird predation in greenhouses has been scant. In companion studies, fire ant workers tended the cotton aphid *A. gossypii* in caged tests and ladybird *C. septempunctata* and *H. convergens* larvae survival was reduced by 84% to 93% on cotton plants infested with *A. gossypii* in the presence of fire ants, as compared to when fire ants were absent [44,77].

Methods of subduing ant aggression against ladybird beetles are necessary to realize the full predation capacity of many ladybird species under pest management regimes in protected plant culture, when pesticides are not used. One possibility could involve using sticky barriers around the periphery and base of the crop plant [78,79]. An alternative, non-physical method could involve releasing ladybird species that do not elicit aggressive behavior in tending or foraging ants. The ability of several ladybird species in the tribe Scymnini, e.g., *Scymnus* species, to forage on aphid-infested plants and reduce aphid populations, even in the presence of tending ants, has been documented in field and laboratory studies [80,81,82]. Chemicals in the waxy covering on the cuticle of *Scymnus* larvae (and other Scymnini) could camouflage them from ants [83]. Aphid-tending ants are likely attracted to a mixture of hydrocarbons on the cuticle of aphids (and other Homoptera) that they tend for honeydew [84]. It is possible that some of the same hydrocarbons on the cuticle of aphids are present on the cuticle and the waxy covering on Scymnini larvae. Aphidophagous ladybird larvae and adults in the tribe Coccinellini (e.g., *Adalia*, *Coleomegilla*, *Harmonia*, and *Coccinella* species) are not camouflaged from ant aggression; they do not have a wax covering. More research is necessary to document the chemical (molecular) basis of this camouflage hypothesis and to manipulate ant-aphid-ladybird densities in greenhouse experiments.

To summarize this section, methods of subduing ant aggression against ladybird beetles on aphid infested plants are necessary to realize the full predation capacity of many ladybird species under organic management regimes in protected plant culture (greenhouses), without deploying pesticides. The predation capacity of some aphidophagous ladybird species (e.g., in the genus *Scymnus*) seems to be undeterred by ants. Little or no research on the efficacy of *Scymnus* species under greenhouse conditions in the presence or absence of aphid-tending ants has been reported, to our knowledge. More research on this topic is necessary.

## 3. Concluding Remarks

This review highlighted the effectiveness of several ladybird species as predators of aphids in greenhouses (and glasshouses). Aphid population reduction exceeded 50% in most studies and ladybird release rates usually did not correlate with aphid reduction. The ratio of aphid reduction/release rate was slightly less for larvae than for adults in some studies, suggesting that larvae were less effective (than adults) in suppressing aphids. Note that some of the adult releases were inside cages, thereby limiting adult dispersion from plants. Overall, the ratio of aphid reduction/release rate was greatest (exceeding a value of 150) for ladybird adults of several species of the normal strain, but least (less than a value of 8) for adults of a flightless *H. axyridis* strain. This may suggest that adults of the normal strain (rather than the flightless strain) were more effective aphid predators in greenhouses.

Based on the limited number of species, and available studies, it was not clear which species was most effective. A comparison of predation capacity, voracity, body size, and tolerance to temperature extremes, amongst species, could help predict which species would be most effective in greenhouses. Many attack a range of aphid species and could be equally effective, if host plant defenses (e.g., leaf trichomes) do not disrupt ladybird foraging behavior. Integrating plant defenses with natural enemies to suppress arthropod pests provides challenges and opportunities [85].

From a practical standpoint, the choice of which species to use may heavily depend on the ease of rearing. For augmentative releases, mass rearing would be necessary to supply the large quantity of high-quality individuals required to reduce aphid densities [86]. Note that *H. axyridis* has been mass-produced and sold commercially for aphid control. Unfortunately, because of the rapid global expansion and negative effects that this species has purportedly had on other ladybirds (through intraguild predation), commercial production and sale of *H. axyridis* has waned. Although a flightless *H. axyridis* strain has been developed and proven effective for aphid control [87,88], it may or may not alleviate concerns of adults escaping from greenhouses and establishing themselves in the neighboring landscape [89].

Because of the limited availability of mass-produced aphidophagous ladybirds currently on the market, a few biocontrol producers and retailers are involved in the acquisition and sale of field-collected, overwintered *H. convergens* adults to growers in North America. Adults are harvested from the Sierra Nevada (western USA) foothills each year [90]. Many of these adults are still in a physiological diapause state upon removal from overwintering sites. As reported previously, *H. convergens* adults have a strong flight propensity and tend to disperse from release sites within a few days [19]. Their effectiveness in reducing aphid populations in greenhouses needs further study. However, releases of field-collected, overwintered *H. convergens* adults, at extremely high release rates, did provide some level of aphid control in experimental nurseries [91].

Intraguild interactions between ladybirds and other predators, as well as aphid parasitoids, requires further study. Nevertheless, this review provides some evidence that intraguild predation does not deter ladybird predation of aphids. More research is necessary to determine if intraguild predation between ladybirds and generalist predators, such as minute pirate bugs, hoverflies, and lacewings (which are also sold by biocontrol companies for aphid control), affects ladybird efficacy under greenhouse conditions. Interestingly, this review also provides evidence that ladybird predation of parasitoids (developing inside aphid prey) often does not hinder the suppression of aphid populations on plants.

Encounters between ladybirds and foraging or aphid-tending ants in greenhouses has not been thoroughly studied. The few available studies clearly reveal that ants hinder most ladybirds, especially larvae, from attacking aphids. Physical barriers will be necessary to curb the entry of ants into greenhouses. Development of alternative methods to reduce ant aggression towards ladybirds on aphid-infested plants is also necessary. In addition, exploring the possibilities of mass producing and deploying ladybirds (e.g., some *Scymnus* species) that do not elicit ant aggression would be a worthwhile line of research.

The conditions for successful aphid control by ladybirds in greenhouses has been reviewed. Despite the challenges, using ladybirds to manage aphid populations in greenhouses and similar protective structures is encouraged.

## Figures and Tables

**Table 1:** Greenhouse studies providing reliable data on ladybird release ratio, rate, and percent aphid reduction within a specified time frame, relative to host plant defenses, life stage, and presence of other predators.

Factors	Ladybird	Aphid	Plant	Release Ratio (L:A)	Release Rate (L/A)	Aphid Reduction (%) ^1^	Time Frame (Days)	Reference
Host plant defenses	*Coccinella septempunctata* (1st instars)	*Aphis fabae*	*Vicia faba*, c.v. major *V. faba*, c.v. 79S4 [in cages]	1:1	1.0	32.8	9	Shannag and Obeidat 2008 [45]
				1:1	1.0	57.1	9	
	*Cycloneda sanguinea* (2nd instars) *Coleomegilla maculata* (2nd instars) *Adalia bipunctata* (2nd instars)	*Aphis gossypii*	*Cucumis sativus*	1:50	0.02	50.45 ^†^	11	Gurney and Hussey 1970 [48]
				1:50	0.02	0 ^†^	11	
				1:50	0.02	36.02 ^†^	11	
	*C. sanguinea* (2nd instars) *C. maculata* (2nd instars) *A. bipunctata* (2nd instars)	*Myzus persicae*	*Chrysanthemum indicum*, c.v. BGA Tuneful	1:100	0.01	99.36 ^†^	07	Gurney and Hussey 1970 [48]
				1:100	0.01	96.64 ^†^	07	
				1:100	0.01	97.77 ^†^	07	
	*A. bipunctata* (adults)	*M. persicae*	*C. indicum* [in cages]	1:117	0.008	73.02 ^†^	14	Gurney and Hussey 1970 [48]
				1:21	0.047	88.85 ^†^	14	
	*C. sanguinea* (adults) *Hippodamia convergens* (adults)	*A. gossypii*	*Gossypium hirsutum*	1:100	0.01	93.5 ^†^	2	Boiça et al. 2004 [49]
				1:100	0.01	86.9 ^†^	2	
Life stage (normal strain)	*C. septempunctata* (1st instars) *A. bipunctata* (1st instars)	*M. persicae*	*Capsicum annuum*	1:10	0.10	93.7 ^†^	10	Hämäläinen 1977 [50]
				1:20	0.05	35.1 ^†^	10	
				1:10	0.10	97.5 ^†^	10	
				1:20	0.05	85.7 ^†^	10	
	*C. septempunctata* (1st instars) *A. bipunctata* (1st instars)	*M. persicae*	*Chrysanthemum morifolium*	1:43	0.023	90.7	08	Hämäläinen 1977 [50]
				1:16	0.062	91.5	08	
				1:75	0.013	77.3	08	
	*C. septempunctata* (adults) *A. bipunctata* (adults)	*M. persicae*	*C. morifolium*	1:12	0.083	86.95	08	Hämäläinen 1977 [50]
				1:50	0.02	94.0	08	
				1:39	0.025	46.15	08	
	*Leis* (*Harmonia*) *dimidiata* (2nd instars)	*A. gossypii*	*C. sativus*	1:10	0.10	85–90	--	Kuznetsov & Hong 2002 [51]
				1:20	0.05	85–90	--	
	*Harmonia axyridis* (3rd, 4th instars)	*Chaetosiphon minor*, *Aphis forbesi*	*Fragaria* × *ananassa*	1:1.73	0.58	84.6	29	Seo & Youn 2002 [52]
	*C. maculata* (3rd instars)	*A. gossypii*	*F*. × *ananassa* [in cages]	1:15	0.07	46.3	14	Rondon et al. 2005 [53]
				1:5	0.20	87.3	14	
				1:3	0.33	96.4	14	
	*H. axyridis* (adults)	*M. persicae*	*C. annuum* [in cages]	1:20	0.05	99.2 ^†^	10	LaRock et al. 2003 [54]
				1:40	0.02	99.4 ^†^	10	
				1:80	0.01	99.5 ^†^	10	
				1:160	0.006	98.9 ^†^	10	
				1:320	0.003	95.3 ^†^	10	
				1:640	0.001	85.7 ^†^	10	
Life stage (flightless strain)	*H. axyridis* (1st instars) (flightless strain)	*A. gossypii*	*C. sativus*	1:2.5	0.4	65.0 ^†^	05	Kuroda & Miura 2003 [55]
				1:0.5	2.0	98.3 ^†^	05	
				1:0.25	4.0	99.5 ^†^	05	
	*H. axyridis* (2nd instars) (flightless strain)	*Lipaphis erysimi*	*Brassica rapa*	1:0.14	7.1	98.0	21	Adachi-Hagimori et al. 2011 [56]
	*H. axyridis* (adults) (flightless strain)	*M. persicae* *L. erysimi*	*B. rapa*	1:3.9	0.25	72.0–98.0	21	Adachi-Hagimori et al. 2011 [56]
				1:3.9	0.25	81.0	21	
	*H. axyridis* (2nd instars) (flightless strain) *H. axyridis* (adults) (flightless strain)	*Aulacorthum solani*	*Solanum melongena*	1:5	0.20	66.7	21	Seko et al. 2014 [57]
				1:25	0.04	66.7	21	
Other predators	*Coccinella transversoguttata* (adults) *C. transversoguttata* (adults) plus one hemipteran *C. transversoguttata* (adults) plus two hemipterans	*M. persicae*	*Beta vulgaris*	1:28.5	0.035	100	6	Tamaki and Weeks 1972 [58]
				1:28.5	0.035	100	6	
				1:28.5	0.035	100	6	

L, Ladybird; A, Aphid. **^1^** Percent aphid reduction calculated using one of two methods: ^†^ When control cages or control greenhouses were used, and aphid density was equilibrated at the onset of the experiment, percent aphid reduction was calculated from aphid density on control and test plants [(control − test)/control (×100)] at post-release evaluation. In the absence of controls, or if aphid density was variable at the onset of the experiment, percent aphid reduction was calculated from aphid density on plants before releasing ladybirds (pre-release) and after releasing them (post-release) [(pre-release − post-release)/pre-release (×100)].
